# Trajectories of the current situation and characteristics of workplace violence among nurses: a nine-year follow-up study

**DOI:** 10.1186/s12913-021-07245-y

**Published:** 2021-11-11

**Authors:** Jianzheng Cai, Ziyu Qin, Haifang Wang, Xiaoqing Zhao, Weixia Yu, Sisi Wu, Ying Zhang, Yalan Wang

**Affiliations:** Department of Nursing, the First Affiliated Hospital of Soochow University, 899 Pinghai Road, Gusu District, 215006 Suzhou, Jiangsu China

**Keywords:** Nurse, Workplace violence, General hospital, Comparison

## Abstract

**Background:**

Workplace violence (WPV) among nurses has become an increasingly serious public health issue worldwide. Investigating the status quo and characteristics of WPV among nurses in different time periods can help hospital managers understand the current status of WPV and its trends over time. This study aimed to understand the current situation of WPV among nurses in Suzhou general hospitals from 2010 to 2019 and analyze changes over time.

**Methods:**

A cross-sectional study was conducted to investigate 942, 2,110 and 2,566 nurses in 6 fixed polyclinic hospitals in Suzhou in 2010, 2015 and 2019, respectively. This study used the revised version of the hospital WPV questionnaire. The count data are described as frequencies and percentages, and the measurement data are represented as means and standard deviations. The general data of nurses during different time periods, the incidence of WPV, nurses’ cognition and attitudes toward WPV and the attitudes and measures of hospitals regarding WPV were analyzed by the chi-square test.

**Results:**

The incidence of WPV among nurses in Suzhou general hospitals in 2015 (69.0 %) and in 2019 (68.4 %) was higher than the incidence of 62.4 % in 2010 (*P*<0.05), and there were significant differences among periods in the specific types of violence (*P*˂0.05). Nurses who participated in the surveys in 2015 and 2019 scored higher on “having heard of WPV before”, “thinking WPV coping management organizations are needed” and “supporting a zero-tolerance policy” than those who participated in 2010 (*P*<0.05). The attitudes and responses of hospitals with regard to WPV among nurses have greatly improved, as evidenced by the results for the items “offering training”, “encouraging reporting of WPV to supervisors”, “equipped with a WPV managing department”, “handling WPV efficiently” and “hospital’s attitudes” (*P*<0.005).

**Conclusions:**

Despite an increase in nurses’ awareness and attitudes regarding WPV and significant improvements in hospitals’ attitudes and responses to WPV, the incidence of WPV remains high. Hospitals should continue to explore scientific training modes that are in accordance with the needs of nurses to reduce the incidence of WPV.

## Background

According to the World Health Organization (WHO), workplace violence (WPV) can be defined as “incidents where staff are abused, threatened or assaulted in circumstances related to their work, involving an explicit or implicit challenge to their safety, well-being or health”. WPV can manifest as physical violence or psychological violence in different forms [1]. In recent years, as the reform of China’s medical system has entered the “deep water zone”, the doctor-patient relationship is becoming increasingly tense, and doctor-patient conflict is gradually emerging. As the main manifestation of doctor-patient conflict, the incidence of hospital WPV is increasing yearly. As the main workforce in medical and health care institutions, nurses have become the main victims of WPV due to the nature of their work, which requires direct contact and frequent communication with patients [2–5]. Studies from outside of China have shown that approximately 70 %-90 % of nurses have experienced one or more types of WPV [6–8]. In China, cross-sectional investigations of WPV among nurses have shown that the incidence of WPV is 71.9 %-84.6 %, which is similar to the incidence reported in other countries [9–11]. These findings show that WPV among nurses has become a common phenomenon in both developing and developed countries.

WPV is a very serious public health problem that not only causes different degrees of physical and psychological injury to nurses but also has negative effects on hospitals [12, 13]. At the individual level, nurses may suffer from varying degrees of physiological harm, such as chronic pain, elevated blood pressure, gastrointestinal disorders, nightmares and even death [14, 15]. Regarding the psychological effects of WPV, in addition to feelings of grievance, anger, anxiety and other emotions, nurses may experience symptoms of insomnia, suicidal ideation and post-traumatic stress disorder [16–18]. At the hospital level, WPV reduces the enthusiasm of nurses toward their work and the quality of their work and increases their turnover rate, which affects the stability of the nursing team [19, 20]. In summary, WPV among nurses is characterized by a high incidence rate and great harmfulness.

Since the 1980 s, scholars at home and abroad have sought to reduce hospital WPV incidence and improve nurses’ ability to respond through a series of measures, including improving medical-related laws and regulations [21], improving the hospital environment and treatment procedures [22], improving nurses’ communication ability [23], and focusing on WPV training [24]. However, a review of the current domestic and foreign literature found that the incidence of WPV among nurses remains high [25, 26]. Some scholars [27] have pointed out that due to the lack of long-term tracking data on the occurrence of WPV among nurses, the existing prevention and intervention measures lack pertinence. A study by Spelten et al. [28] pointed out that reducing the incidence of WPV first requires a comprehensive understanding of the dynamic development process of violence toward nurses, including by determining the frequency and intensity of WPV events and assessing the long-term results of violence on staff. However, most of the existing WPV studies are short-term status surveys, and few studies continuously compare the WPV status and characteristics of nurses in a certain region. This study aimed to analyze the current situation and characteristics of WPV among nurses in Suzhou general hospitals from 2010 to 2019 to provide information that can help hospital managers understand the status quo of WPV among nurses and trends in WPV over time.

## Methods

### Study design

In this cross-sectional study, structured questionnaires were used to investigate the occurrence of WPV among nurses in 6 general hospitals in Suzhou in 2010, 2015 and 2019.

### Participants

A total of 942, 2,110 and 2,566 clinical nurses were selected from Suzhou general hospitals in November of each year in 2010, 2015 and 2019, respectively, by the convenience sampling method.

### Inclusion criteria

(1) Employment as in-service nurses in clinical departments who had direct contact with patients in their daily work.

(2) Professional certification.

(3) Completion of at least 1 year of clinical nursing work.

 (4) Provision of a signed informed consent form and voluntary participation in the study.

### Exclusion criteria

(1) Nurses off-duty during the investigation, e.g., on maternity leave, sick leave, or vacation.

(2) Non-regular nurses and personnel undergoing refresher training in our hospital.

### Ethics approval

 The study was approved by the Medical Ethics Committee of the First Affiliated Hospital of Soochow University in China (No. 2,018,062). The participants were made aware of the aim and procedures of the study, and they all signed an informed consent form. The researchers guaranteed the confidentiality of the subjects’ information and that it would be used only for research purposes. The voluntary principle was always followed during the study, and the participants could leave the study at any time.

## Measurement instruments

### General information questionnaire

The questionnaire was designed by the research group to suit the research purpose and collected information on gender, age, education, whether an only child, marital status, hospital grade, department, years worked, employment type and professional title.

### Revised version of the hospital WPV questionnaire

The questionnaire designed by Shengyong Wang [29] of Jinan University includes 33 entries in 4 dimensions: the frequency of WPV occurrence in the past year, the most impressive WPV experience in the past year, cognition and attitudes toward WPV, and the attitudes and responses of hospitals to WPV. The questionnaire has been widely used in clinical practice. The questionnaire has good reliability and validity; the test-retest reliability of the questionnaire was 0.803, and the average content validity of each item was 0.916.

### Data collection

Two weeks before the beginning of the study, the head of the research team (which included 2 postgraduate tutors, 6 postgraduates, and 4 undergraduates) contacted the managers of each hospital by telephone or e-mail to request the support of the corresponding person in charge for the study and published a notice on each hospital website to recruit participants to participate in the survey. The contents of the notice included the location, time, purpose, significance, confidentiality principle and precautions of the questionnaire. If nurses were willing to participate in the survey, they could go to a designated conference room to receive the questionnaire and complete it anonymously. Each hospital was equipped with 2 research team members. If a nurse had any questions during completion of the questionnaire, the project team members could give guidance at any time. After completing the questionnaire, the nurse placed it into the questionnaire recovery box in the conference room. At 6:00 p.m., after the nurses had completed the questionnaires, the questionnaire recovery box was sealed by the members of the research group and sent to the questionnaire archives of the research group for safekeeping.

### Statistical analysis

Data management and analysis were performed using SPSS 18.0. The count data are described as frequencies and percentages, and the quantitative data are reported as means and standard deviations. The chi-square test was used to analyze the general data of the nurses from the different time periods, the incidence of WPV, nurses’ cognition and attitudes regarding WPV and the hospitals’ attitudes toward WPV and corresponding measures. The Mann-Whitney U test was used to analyze the demographic data. Statistical significance was set at *P*<0.05.

## Results

### Questionnaire response rate

In 2010, 2015 and 2019, 1,000, 2,132 and 2,700 questionnaires were distributed, respectively, and 942, 2,110 and 2,566 effective questionnaires were actually recovered, for effective response rates of 94.20 %, 98.97 % and 95.04 %.

### Nurses’ general information

A total of 5,618 nurses completed the three surveys, including 388 emergency nurses, 618 outpatient nurses and 4,612 ward nurses. The age of the respondents ranged from 20 to 63 years, with an average age of 31.41 ± 5.82 years, and the proportion of respondents < 30 years old was 51.6 %. The number of years worked ranged from 1 to 37, with the average being 8.85 ± 7.45 years. Of the respondents, 23.2 % had worked ≤ 5 years; 52.8 % had a bachelor’s degree; and 42.7 % had junior professional titles. The demographic characteristics are presented in Table 1.

**Table 1:** Comparison of the demographic characteristics of the nurses, n (%)

Items	Categories	2010	2015	2019	*χ*^2^*/Z*	*P*
Age (Years)	<30	506 (53.7)	1080 (51.2)	1311 (51.1)	16.915^*^	0.010
	30~39	348 (36.9)	737 (34.9)	933 (36.4)		
	40~49	75 (8.0)	236 (11.2)	278 (10.8)		
	≥50	13 (1.4)	57 (2.7)	44 (1.7)		
Gender	Female	894 (94.9)	2049 (97.1)	2396(93.4)	34.249	<0.001
	Male	48 (5.1)	61 (2.9)	170(6.6)		
Marital status	Married	605 (64.2)	1430 (67.8)	1628 (63.4)	17.257	0.002
	Single	337 (35.8)	665 (31.5)	922 (35.9)		
	Other	0 (0.0)	15 (0.7)	16 (0.6)		
Only child	Yes	392 (41.6)	1098 (52.0)	1291 (50.3)	29.551	<0.001
	No	550 (58.4)	1012 (48.0)	1275 (49.7)		
Education level	Technical secondary degree or below	62 (6.6)	63 (3.0)	33 (1.3)	385.760^*^	<0.001
	Junior college	568 (60.3)	1008 (47.8)	811 (31.6)		
	Bachelor’s degree	305 (32.4)	1019 (48.3)	1656 (64.5)		
	Master’s degree or above	7 (0.7)	20 (0.9)	66 (2.6)		
Years worked	≤5	194 (20.6)	381 (18.1)	726 (28.3)	85.897^*^	<0.001
	6~10	294 (31.2)	775 (36.7)	847 (33.0)		
	11~15	204 (21.7)	384 (18.2)	428 (16.7)		
	≥16	250 (26.5)	570 (27.0)	565 (22.0)		
Professional title	Senior	324 (34.4)	687 (32.6)	592 (23.1)	120.281^*^	<0.001
	Medium	288 (30.6)	640 (30.3)	688 (26.8)		
	Junior	330 (30.5)	783 (37.1)	1286 (50.1)		
Post	Nurse	901 (95.6)	1977 (93.7)	2432 (94.8)	5.401	0.067
	Head nurse	41 (4.4)	133 (6.3)	134 (5.2)		
Employment type	Official staff	495 (52.5)	1068 (50.6)	1213 (47.3)	9.878	0.043
	Contract staff	437 (46.4)	1016 (48.2)	1317 (51.3)		
	Temporary staff	10 (1.1)	26 (1.2)	36 (1.4)		
Department	Emergency	59 (6.3)	168 (8.0)	161 (6.3)	77.396	<0.001
	Outpatient	159 (16.9)	265 (12.6)	194 (7.6)		
	Ward	724 (76.9)	1677 (79.5)	2211 (86.2)		

^*^: The Mann-Whitney U test was used

### Incidence of WPV among nurses in 2010, 2015, and 2019

The incidence of WPV among nurses was 62.4 % in 2010, 69.0 % in 2015 and 68.4 % in 2019 (*P*=0.001). Compared with that in 2010, the incidence increased significantly in 2015 and 2019 (*P*<0.001, *P*=0.001). The details are shown in Table 2.

**Table 2:** Comparison of the incidence of WPV among nurses in different years, n (%)

Year	Quantity	Not exposed to WPV	Exposed to WPV	*χ*^*2*^	*P*
T_1_ (2010)	942	354 (37.6)	588 (62.4)	13.979	0.001
T_2_ (2015)	2110	655 (31.0)	1455 (69.0)		
T_3_ (2019)	2566	812 (31.6)	1754 (68.4)		
Multiple comparison					
*P*_T1−T2_				12.575	<0.001
*P*_T1−T3_				10.938	0.001
*P*_T2−T3_				0.195	0.681

### Incidence of different forms of WPV among nurses in 2010, 2015, and 2019

According to the current situation of WPV toward nurses in different years, the proportion of nurses who experienced sexual harassment (4.1 %) was the highest in 2010. The proportion of people who suffered verbal abuse (66.2 %) and threats (46.3 %) was the highest in 2015. Physical assault (13.1 %) was the highest in 2019. The overall comparison of WPV in different years and of different types showed significant differences (*P*<0.05). Further multiple comparisons of the data showed that there were highly statistically significant differences between the total number of WPV incidents in 2010 and 2015 and between the number of physical assault incidents in 2015 and 2019 (*P*<0.001). The details are shown in Table 3

**Table 3:** Comparison of different forms of WPV among nurses in different years, n (%)

Year		Quantity	Exposed to WPV	Verbal abuse	Verbal threat	Physical assault	Sexual harassment
T_1_ (2010)		942	588 (62.4)	570 (60.5)	387 (41.1)	93 (9.9)	39 (4.1)
T_2_ (2015)		2110	1455 (69.0)	1396 (66.2)	977 (46.3)	211 (10.0)	55 (2.6)
T_3_ (2019)		2566	1754 (68.4)	1651 (64.3)	1116 (43.5)	335 (13.1)	102 (4.0)
*χ*^*2*^			13.979	28.588	19.423	26.865	16.185
*P*			0.001	<0.001	0.004	<0.001	0.013
Multiple comparison							
*P*	T_1_-T_2_		<0.001	0.012	0.440	0.232	0.025
	T_1_-T_3_		0.001	0.005	0.250	0.048	0.303
	T_2_-T_3_		0.681	0.001	0.020	<0.001	0.010

### Cognition and attitudes of nurses toward WPV in 2010, 2015 and 2019

Compared with the number in 2010, more nurses surveyed in 2015 and 2019 said that they had heard of WPV before, and the result was considered statistically significant (*P*_T1−T2_=0.020, *P*_T1−T3_=0.032). In 2010 and 2015, less about WPV was included in the nurses’ preservice training (*P*_T1−T3_=0.002, *P*_T2−T3_=0.001), but more nurses thought they would benefit from WPV training (*P*_T1−T3_=0.016, *P*_T2−T3_=0.010). The study also found that clinical nurses in 2015 and 2019 were more likely than nurses in 2010 to approve of hospitals setting up WPV processing facilities and to support a zero-tolerance policy toward WPV (*P*<0.001). In addition, more than 85 % of nurses involved in the three surveys expressed their willingness to attend WPV training and thought they would benefit from it. The details are shown in Table 4

**Table 4:** Comparison of cognition and attitudes toward WPV among nurses in different years, n (%)

Items	Categories	2010	2015	2019	*χ*^*2*^	*P*	*P*		
		(T_1_)	(T_2_)	(T_3_)			T_1_-T_2_	T_1_-T_3_	T_2_-T_3_
Having heard of WPV before	Yes	763 (81.0)	1782 (84.5)	2157 (84.7)	6.166	0.046	0.020	0.032	0.717
	No	179 (19.0)	328 (15.5)	916 (16.3)					
WPV is inevitable at work	Yes	712 (75.6)	1594 (75.5)	1962 (76.5)	0.625	0.732	1.000	0.591	0.470
	No	230 (24.4)	516 (24.5)	604 (24.0)					
Thinking WPV is not worth the fuss	Yes	132 (14.0)	264 (12.5)	374 (14.6)	4.258	0.119	0.268	0.704	0.044
	No	810 (86.0)	1846 (87.5)	2192 (85.4)					
Thinking preservice training should include training programs on WPV	Yes	922 (97.9)	2058 (97.5)	2457 (95.8)	16.192	<0.001	0.608	0.002	0.001
	No	20 (2.1)	352 (2.5)	109 (4.2)					
Willing to attend the WPV training	Yes	874 (92.8)	1968 (93.3)	2405 (93.7)	1.083	0.582	0.642	0.317	0.551
	No	68 (7.2)	142 (6.7)	151 (6.3)					
Thinking training on WPV would be beneficial	Yes	822 (87.3)	1851 (87.7)	2313 (90.1)	9.279	0.010	0.722	0.016	0.010
	No	120 (12.7)	259 (12.3)	253 (9.9)					
Thinking WPV coping management organizations are needed	Yes	903 (95.9)	2071 (98.2)	2519 (98.2)	19.082	<0.001	<0.001	<0.001	1.000
	No	39 (4.1)	39 (1.8)	47 (1.8)					
Supporting a “zero-tolerance” policy	Yes	618 (65.6)	1882 (89.2)	2310 (90.0)	368.750	<0.001	<0.001	<0.001	0.360
	No	324 (34.4)	228 (10.8)	256 (10.0)					

### Attitudes and responses of hospitals to WPV towards nurses in 2010, 2015 and 2019

This study found that in the past 9 years, the hospitals’ attitudes and responses to WPV towards nurses have greatly improved, as indicated by improvements in five aspects, namely, “offering training”, “encouraging reporting of WPV to supervisors”, “equipped with a WPV management department”, “handling WPV efficiently”, and “hospital’s attitudes” (*P*<0.001). However, most hospitals still have not developed training. Regarding hospitals’ emphasis on WPV, hospitals paid more attention to WPV toward physicians (*P*=0.013). The details are shown in Table 5

**Table 5:** Comparison of attitudes and responses of hospitals toward WPV among nurses in different years, n (%)

Items	Categories	2010	2015	2019	*χ*^*2*^	*P*	*P*		
		(T_1_)	(T_2_)	(T_3_)			T_1_-T_2_	T_1_-T_3_	T_2_-T_3_
Offering training	Yes	91 (9.7)	487 (23.1)	840 (32.7)	202.794	<0.001	<0.001	<0.001	<0.001
	No	851 (90.3)	1623 (76.9)	1726 (67.3)					
Encouraging reporting of WPV to supervisors	Yes	585 (62.1)	1575 (74.6)	1849 (72.1)	51.255	<0.001	<0.001	<0.001	0.050
	No	357 (37.9)	535 (25.4)	717 (27.9)					
Equipped with WPV management department	Yes	432 (45.9)	1215 (57.6)	1451 (56.5)	39.942	<0.001	<0.001	<0.001	0.495
	No	510 (54.1)	895 (42.4)	1115 (43.5)					
Handling WPV efficiently	Yes	264 (28.0)	791 (37.5)	1193 (46.5)	106.884	<0.001	<0.001	<0.001	<0.001
	No	678 (72.0)	1319 (62.5)	1373 (53.5)					
Hospital’s attitudes	Protecting the interests of staff	91 (9.7)	574 (27.2)	775 (30.2)	283.785	<0.001	<0.001	<0.001	<0.001
	Handling WPV fairly based on the facts	425 (45.1)	855 (40.5)	1194 (46.5)					
	Turning a blind eye	283 (30.0)	549 (26.0)	470 (18.3)					
	Punishing staff regardless of the cause	143 (15.2)	132 (6.3)	127 (4.9)					
Degree of emphasis	Physician>Nurse	403 (42.8)	959 (45.5)	1117 (43.5)	12.649	0.013	0.002	<0.001	0.346
	Nurse>Physician	0 (0.0)	22 (1.0)	23 (0.9)					
	Physician=Nurse	539 (57.2)	1129 (53.5)	1426 (55.6)					

## Discussion

This study found that the overall incidence of WPV towards nurses in Suzhou general hospitals gradually increased from 62.4 to 68.4 % from 2010 to 2019 and significantly increased from 2010 to 2015. There were significant differences at first (*P*<0.05), but then a plateau was reached (*P>*0.05). The incidence reported here is lower than the overall WPV incidence of 76 % among 762 registered nurses identified in a study from the U.S. [30], higher than the incidence reported in a study conducted in Botswana (44.1 %) [31], and similar to the incidence reported in a study by Ren et al. (68.1 %) [32]. The incidence of WPV towards nurses varies by country, region, social background and sample size. However, the phenomenon of WPV against nurses has become increasingly common at home and abroad, in developing countries and developed countries. A comparison of the types of WPV nurses experience across the three study years showed that “verbal abuse” is the most common form of WPV, which is consistent with most surveys [33–36]. Verbal abuse and threats showed an upward trend and then a downward trend from 2010 to 2019, with the highest incidence in 2015. This was possibly related to the greater emphasis of hospitals on the training of nurses’ service attitudes and communication skills in recent years. To reduce the incidence of verbal abuse or threats, hospital management developed training on coping and communication strategies for nurses, including using amiable words, putting themselves in patients’ shoes and listening to the needs of patients from the patients’ point of view so that patients could feel understood and respected. However, the lack of corresponding legal constraints on verbal violence led to nurses being unable to obtain timely evidence and a fair hearing after experiencing this type of WPV, which made verbal violence hard to intervene in and prevent. The results of this survey showed that the incidence of physical assault against nurses has gradually increased over the past nine years. Though the incidence of physical violence was relatively low between 2010 and 2015 (*P>*0.05), the incidence of physical assault increased significantly from 2015 to 2019 (*P*<0.001). With the development of the economy and the improvement of living standards, people’s demand for medical treatment has changed; people are not satisfied with only cure of the disease itself but are increasingly attentive to the overall effect of treatment and attach increasing importance to the experience of the treatment process. When patients and their relatives have unpleasant experiences or their requirements are not met during medical treatment, it is easy for conflicts to arise between nurses and patients. If the hospital treatment system or process is not perfect, such conflict is likely to escalate and induce physical aggression. It is recommended that hospital management give attention to the various forms of WPV while focusing on the service attitude, the patient’s medical experience and the transformation of the service process. Hospital management should focus on not only implementing policies but also establishing hospital security forces and an effective cooperation mechanism between hospitals and public security systems, implementing measures such as increasing patrol times, improving security inspections, prohibiting the carrying of sharp or dangerous goods into hospitals and developing security training for staff [37]. Such practices would allow a hospital to intervene immediately in the event of violence, ensure the safety of medical personnel and reduce the occurrence of violence through multi-sector coordination. It was shown that the incidence of sexual harassment among nurses had a decreasing trend and then an increasing trend over time, with the incidence being basically flat in 2010 and 2019, which was due to the improvement of laws and the health care system in recent years. Since 2014, China has issued a series of laws and regulations, such as the Regulations on the Prevention and Treatment of Medical Disputes, the Memorandum of Cooperation on Joint Punishment of Persons who seriously endanger the Normal Medical Order, and the Basic Medical and Health Promotion Law, which have played a positive role in protecting legitimate rights by law, deterring illegal acts targeting medical staff and institutions and maintaining the order of hospitals. However, with the passage of time, some law enforcement officers have not truly implemented the laws and regulations, resulting in a rebound trend of hospital WPV since 2015. Therefore, it is suggested that law enforcement officers should sternly punish people who attack medical staff and promote the implementation of policies and laws so that they can effectively protect the legitimate rights and interests of nurses.

In the past 9 years, nurses’ cognition and attitudes toward WPV have improved. The rates of “having heard of WPV before”, “thinking WPV coping management organizations are needed” and “supporting a zero-tolerance policy” increased significantly from 2010 to 2015 (*P*<0.05) but showed no great changes from 2015 to 2019 (*P*>0.05), which was likely related to the formulation of corresponding preventive measures and provision of reasonable and effective education and training. The National Institute of Occupational Safety and Health [38] developed and delivered WPV prevention training course for nurses, and more than 90 % of participants thought the course filled gaps in their knowledge. It was also found that the reasons why some nurses were unable to effectively judge whether they had experienced WPV were that they lacked an understanding of the concept of WPV or had never heard the term WPV before. Ming et al. [39] found that after a randomized intervention for clinical nurses, the cognition and attitudes of the nurses who participated in violence training were higher than those who did not. Brann et al. [40] found that the level of cognition and knowledge of WPV among nurses was higher than before training; nurses could identify violence that they had experienced personally or as bystanders and no longer considered violence part of their work. Therefore, it is necessary to develop training on WPV for clinical nurses. In addition, the results of the three surveys showed that although the majority of nurses were willing to participate in WPV prevention training, there was less WPV content in their preservice training in 2019 than in 2010 and 2015 (*P*_T1−T3_=0.002, *P*_T2−T3_=0.001), which indicates to hospital management that in order to maximize the effect of education and training and improve nurses’ ability to identify and deal with WPV in clinical practice, it is necessary to strengthen the training on WPV and make it more systematic and routine, integrate WPV content into new nurses’ education and in-hospital education, regularly disseminate knowledge on violence to nurses, strengthen continuous management after the training, and promote the continuous improvement of the post-training results while meeting the learning needs of nurses [41]. Furthermore, it is necessary to carry out public education, publicize the key role of the hospital in solving problems in the medical system through various networks, and increase public understanding of the efforts of medical centers to improve the medical experience and providing services to patients to decrease WPV.

In this survey, hospitals’ attitudes and responses to nurses’ WPV improved significantly from 2010 to 2019, which was embodied in five aspects: “offering training”, “encouraging reporting of WPV to supervisors”, “equipped with a WPV management department”, “handling WPV efficiently” and “hospital’s attitudes” (*P*<0.05). The differences in the response rates for “offering training”, “handling WPV efficiently”, “hospital’s attitudes” at different times were statistically significant (*P*<0.001), which may be related to changes in the management philosophy of the hospital managers. At present, hospital management pays more attention to the safety of nurses, constantly explores and develops scientific and effective nurse-related WPV management measures, actively and effectively controls violence, addresses nurses’ suffering from violence and minimizes hospital violence caused by improper or unclear management policies [37]. However, less than half of respondents reported receiving WPV prevention-related training. Although the training incidence increased in 2019 compared with that in 2010 and 2015, it was only 32.7 %, which is consistent with the results of other studies [42, 43]. The results of the survey also showed that more than 95 % of nurses believed that preservice training should include training on WPV, and more than 90 % of nurses expressed a willingness to attend, which showed that the current hospital-related education and training is far from meeting the needs of clinical nurses. To reduce the incidence of WPV or minimize the harm of WPV, nurses’ WPV management intervention skills should be strengthened, with special attention given to developing training, exploring the content and form of training, increasing the initiative and enthusiasm of nurses, and improving nurses’ cognition, attitudes and coping abilities with regard to WPV. In addition, compared with the results from 2010, those from 2015 to 2019 showed that hospitals paid more attention to doctors who had experienced WPV, with a gradual upward trend (*P*<0.001). Some studies [19, 44, 45] suggest that organizational support and hospital management can reduce the negative effects of WPV on nurses. Without strong hospital attention, nurses may lack trust in the management department and experience reduced job satisfaction, which will aggravate adverse reactions to WPV. In conclusion, hospitals should treat WPV among doctors and nurses equally so that nurse-directed WPV can be resolved equally and fairly and the psychological gap of employees can be reduced.

## Conclusions

The incidence of WPV among nurses in Suzhou general hospitals increased in 2015 and 2019 compared to that in 2010. The main form of WPV is verbal abuse. Despite the continuous improvement of nurses′ awareness of WPV, there is still room for improvement in hospitals′ attitudes and responses to WPV, especially in terms of actively carrying out WPV training. In conclusion, hospital managers should aim to comprehensively understand the dynamics of WPV, especially the trends of violence over long time periods, to reduce the incidence of WPV among nurses.

## Data Availability

Due to further data analysis is in progress, and the research group reached an agreement with the nurses and hospital management managers to restrict data sharing, the datasets generated and analyzed during the current study are only used for this study and cannot be publicly available. However, under reasonable requirements, the data and material of this study can be obtained from the corresponding author.

## Abbreviations

WHO: World Health Organization
WPV: Workplace violence
